# Analysis of transcriptome data and quantitative trait loci enables the identification of candidate genes responsible for fiber strength in *Gossypium barbadense*

**DOI:** 10.1093/g3journal/jkac167

**Published:** 2022-07-26

**Authors:** Yajie Duan, Qin Chen, Quanjia Chen, Kai Zheng, Yongsheng Cai, Yilei Long, Jieyin Zhao, Yaping Guo, Fenglei Sun, Yanying Qu

**Affiliations:** College of Agronomy, Xinjiang Agricultural University, Urumqi, Xinjiang 830052, China; College of Agronomy, Xinjiang Agricultural University, Urumqi, Xinjiang 830052, China; College of Agronomy, Xinjiang Agricultural University, Urumqi, Xinjiang 830052, China; College of Agronomy, Xinjiang Agricultural University, Urumqi, Xinjiang 830052, China; College of Agronomy, Xinjiang Agricultural University, Urumqi, Xinjiang 830052, China; College of Agronomy, Xinjiang Agricultural University, Urumqi, Xinjiang 830052, China; College of Agronomy, Xinjiang Agricultural University, Urumqi, Xinjiang 830052, China; College of Agronomy, Xinjiang Agricultural University, Urumqi, Xinjiang 830052, China; College of Agronomy, Xinjiang Agricultural University, Urumqi, Xinjiang 830052, China; College of Agronomy, Xinjiang Agricultural University, Urumqi, Xinjiang 830052, China

**Keywords:** *Gossypium barbadense*, fiber strength, RNA-seq, weighted gene coexpression network analysis, QTL, MPP, Multiparental Populations, Multiparent Advanced Generation Inter-Cross (MAGIC)

## Abstract

*Gossypium barbadense* possesses a superior fiber quality because of its fiber length and strength. An in-depth analysis of the underlying genetic mechanism could aid in filling the gap in research regarding fiber strength and could provide helpful information for *Gossypium barbadense* breeding. Three quantitative trait loci related to fiber strength were identified from a *Gossypium barbadense* recombinant inbred line (PimaS-7 × 5917) for further analysis. RNA sequencing was performed in the fiber tissues of PimaS-7 × 5917 0–35 days postanthesis. Four specific modules closely related to the secondary wall-thickening stage were obtained using the weighted gene coexpression network analysis. In total, 55 genes were identified as differentially expressed from 4 specific modules. Gene Ontology and the Kyoto Encyclopedia of Genes and Genomes were used for enrichment analysis, and *Gbar_D11G032910*, *Gbar_D08G020540*, *Gbar_D08G013370*, *Gbar_D11G033670*, and *Gbar_D11G029020* were found to regulate fiber strength by playing a role in the composition of structural constituents of cytoskeleton and microtubules during fiber development. Quantitative real-time PCR results confirmed the accuracy of the transcriptome data. This study provides a quick strategy for exploring candidate genes and provides new insights for improving fiber strength in cotton.

## Introduction

*Gossypium* *barbadense*, native to South America, is one of the 4 cultivated *Gossypium* species. *G. barbadense* has the best fiber quality, and its fiber length, fineness, strength, tensile strength, and other characteristics are better than that of other cotton varieties. Due to low self-production and limited areas for the cultivation of *G. barbadense*, the main production areas in China are highly limited, and the yield accounts for only 2% of the total cotton production (Chen *et al.* 2007). Furthermore, these factors limit further study of *G. barbadense* fiber quality traits. Fiber strength (FS) is a quantitative trait controlled by multiple genes (Tu *et al.* 2007; Shi *et al.* 2015). Hence, an in-depth analysis of the genetic mechanisms affecting FS is necessary; this could aid in filling the gap in research regarding FS and could provide helpful information for improvements in *G. barbadense* breeding.

The process of cotton fiber development includes 4 stages: fiber initiation 0–3 days postanthesis (DPA), fiber elongation (3–23 DPA), secondary wall-thickening (20–40 DPA), and fiber maturity (40–50 DPA). Although there is clear demarcation between the stages, each stage has its characteristic features (Basra and Malik 1984; Haigler *et al.* 2012). Different fiber development stages are used to study different fiber quality traits. For example, fiber initiation and elongation usually affect the fiber length, while FS is more influenced by the secondary wall-thickening stage (Lee *et al.* 2007; Chen and Burke 2015; Chen *et al.* 2019; Patel *et al.* 2020). The main components of cotton fiber are cellulose, hemicellulose, and lignin. Cellulose accumulation and structure are important factors affecting FS (Edelmann and Fry 1992; Shang *et al.* 2016). Cellulose content begins to increase 15 DPA (Potikha *et al.* 1999). During the secondary wall-thickening stage in cotton, as cellulose biosynthesis increases rapidly, complex signaling pathways related to FS, such as signal transduction (Gao *et al.* 2019), metabolic pathways (Li *et al.* 2016; Ahmed *et al.* 2020; Zhao, Dong, *et al.* 2020), and microtubule (Pydiura *et al.* 2019; Chen *et al.* 2021), cellulose, and cytoskeleton pathways are initiated (Tuttle *et al.* 2015; Zhang *et al.* 2015; Sugiyama *et al.* 2017). Therefore, analysis of molecular mechanisms underlying the development of FS is mainly focused on the secondary wall-thickening stage.

With the completion of sequencing for genomes of *G*ossypium *raimondii* (Wang *et al.* 2012), *Gossypiumarboreum* (Li *et al.* 2014), *Gossypiumhirsutum* (Hu *et al.* 2019), and *G. barbadense* (Wang *et al.* 2019), more complete reference genomes are available to study the quantitative traits of cotton. Through analysis of quantitative trait loci (QTLs), numerous QTLs and candidate genes associated with fiber quality were discovered, and the number of QTLs related to FS was found to be second only to those related to fiber length (Ali *et al.* 2018; Fan *et al.* 2018; Keerio *et al.* 2018; Tan *et al.* 2018; Zhang *et al.* 2019; Shi *et al.* 2020; Guo *et al.* 2021). Most stable QTLs related to FS were mapped using the recombinant inbred lines of *G. hirsutum* (Gu *et al.* 2020). *qFS-Chr.D02* was fine-mapped using a single segment introgression line (IL-D2-2 × TM-1) of *G. hirsutum* (Feng *et al.* 2020). Thirty-four stable QTLs associated with 5 fiber quality traits were mapped using 279 accessions of *G. barbadense*, and 6 novel QTLs were mapped using 276 accessions of *G. hirsutum* (Liu *et al.* 2020; Su *et al.* 2020). However, the number of effective molecular markers and major genes did not increase significantly. The different populations and environmental factors make it difficult to find stable QTLs. Based on the reference genome, the obtained QTL confidence interval usually covers a large physical location and includes numerous genes. Thus, it is difficult to identify genes associated with fiber quality.

With the continuous advancements in high-throughput sequencing technology, transcriptome sequencing (RNA-seq) and molecular markers developed using this technology have profoundly changed the strategies and approaches of cotton breeding (Wang, Wang, *et al.* 2020). Transcriptome technology effectively uses changes in gene expression to identify the genes closely related to target traits (Qin *et al.* 2019; Shahzad *et al.* 2020). Weighted gene coexpression network analysis (WGCNA), based on transcriptome data that could identify specific modules and genes related to target traits, was performed by dividing genes with similar expression patterns into the same module (Langfelder and Horvath 2008; Zou *et al.* 2019; Ali *et al.* 2020; Cheng *et al.* 2020). This method has been used to study high-quality fiber in upland cotton, soybean seed set and size, seed development in chickpea, and bacterial spot resistance in pepper in the past 5 years (Du *et al.* 2017; Garg *et al.* 2017; Zou *et al.* 2019; Feng *et al.* 2021; Jiang *et al.* 2021; Zhu, Gao, *et al.* 2021). The combined analysis of the transcriptome and QTLs can quickly narrow down the range of genes and accurately select genes during key developmental stages (Khattak *et al.* 2019; Wang, Zheng, *et al.* 2020; Laoue *et al.* 2021; Li *et al.* 2021). Nine genes in rapeseed were identified as candidate genes related to pod number variation (Ye *et al.* 2017). *KR*, *C4H*, and *FatA* were identified as candidates related to capsaicinoid biosynthesis (Park *et al.* 2019). *OsSAP16* was identified as a candidate related to salt tolerance (Lei *et al.* 2020). However, most studies focused on fiber length traits in the fiber initiation and fiber elongation stages, and thus, there is a lack of further research on FS during the secondary wall-thickening stage (Zhu, Stiller, *et al.* 2021).

PimaS-7 × 5917 was a recombinant inbred line population with 143 individuals constructed in 2013 to study FS traits (Fan *et al.* 2018). In the present study, the confidence intervals of 3 QTLs related to FS traits in PimaS-7 × 5917 population were further analyzed and their corresponding physical locations were obtained. Then, differential expression of 2 (from PimaS-7 × 5917) during whole fiber development was analyzed by transcriptome technique. A weighted gene coexpression network was constructed to screen out specific modules related to FS and combine the genes related to FS based on QTLs to screen and confirm the candidate genes. This study provides a more rapid strategy for shortening the acquisition time of target candidate genes and novel insights for studying genetic basis of cotton FS traits.

## Materials and methods

### Plant material

The RIL population PimaS-7 × 5917 developed using American cotton PimaS-7 and Chinese *G. barbadense* cultivar 5917 was first used to study FS and more construction processes in 2018 (Fan *et al.* 2018). From 2016 to 2018, the RIL population was planted in Aksu (Xinjiang Province, 41°17ʹ07.12 N, 80°26ʹ50.68E). Eight *G. barbadense* accessions (4 with high-FS and 4 with low-FS) were also planted. The experiments were conducted using a randomized group test design (double-row plots, length 5 m, width 0.8 m). All plant materials (provided by the Key Laboratory of Agricultural Biotechnology, Xinjiang Agricultural University, Urumqi, China) were considerably different in FS. The quality of fully mature fiber was determined by the Cotton Fiber Quality Supervision and Testing Center of the Chinese Ministry of Agriculture (HVI1000).

Two *G. barbadense* plants from the parent varieties PimaS-7 and 5917 were used for RNA-seq. Eight *G. barbadense* accessions were planted as verification materials. The day of flowering was recorded as 0 DPA at intervals of 5 days. Cotton fiber samples (3 biological replicates) were collected at 0 DPA (ovules), 5, 10, 15, 20, 25, 30, and 35 DPA. All samples were placed on ice packs and quickly separated with sterilized tweezers and transported in liquid nitrogen. Some samples were used for RNA-seq, while others were used for quantitative real-time PCR (qRT-PCR) and stored at −80°C.

### QTL region realignment

According to Fan’s studies, using single nucleotide polymorphism (SNP) markers, 3 QTLs related to FS were identified from the *G. barbadense* RIL population. To further analyze the 3 QTLs, we extracted SNP marker sequences corresponding to these QTLs (Fan *et al.* 2018). The marker sequences were aligned to the *G. barbadense* genome (Wang *et al.* 2019) using BWA software (Li and Durbin 2009); the physical location of the markers was obtained, and the genes in the corresponding chromosome position were extracted for further analysis.

### RNA extraction, library construction, and RNA-seq

An RNA prep pure plant kit (Tiangen, Beijing, China) was used for the total RNA extraction of all samples. RNA integrity was confirmed using 1% agarose gel electrophoresis. The concentration and the quality of RNA were determined using a Colibri Microphotometer (Titertek Berthold, Germany). A total of 48 cDNA libraries were constructed using an Illumina HiSeq2500 platform (Biomarker Technologies, Beijing, China) for the 2 *G. barbadense* parents at 8 developing times with 3 biological replicates. All RNA-seq raw data were deposited in the NCBI database with the accession number: GSE178945, at https://www.ncbi.nlm.nih.gov/geo/query/acc.cgi?acc=GSE178945.

### Data processing and differentially expressed gene analysis

The raw data obtained by RNA-seq were provided in FASTQ format. Quality control of raw reads was performed by removing adapter sequences, low-quality reads, and reads with *N* > 10% (Bolger *et al.* 2014). The *G. barbadense* Pima 3-79 genome was designated as the reference genome, which can be found at http://cotton.hzau.edu.cn/EN/data/download/G.barbadensegenomeHAU\v2.0.tar (Wang *et al.* 2019). Reads were aligned using HISAT2 (Kim *et al.* 2015) and were assembled and quantified by performing a String Tie (Pertea *et al.* 2015). The fragments per kilobase per million (FPKM) parameter refers to the number of reads per thousand bases from the map to exon per million reads. When the gene expression satisfied FPKM >0.5, the genes were identified to be expressed genes (Mortazavi *et al.* 2008). The DESeq2 R package (v.3.6.3) was used to identify differentially expressed genes (DEGs). When gene expression satisfied the false discovery rate (FDR) value ≤0.05 and |Log2-fold change (FC) | ≥ 1, it was considered to be a DEG (Patel *et al.* 2020). All DEGs were annotated using Gene Ontology (GO) functional enrichment analysis. KOBAS software was used for the Kyoto Encyclopaedia of Genes and Genomes pathway enrichment analysis for all DEGs (Altschul *et al.* 1990; Wu *et al.* 2006; Xie *et al.* 2011).

### Construction of coexpression modules using WGCNA

Coexpression modules were constructed using the WGCNA (v.1.51) package in R (v.3.6.3) (Langfelder and Horvath 2008). In the present study, for filtering out the DEGs with low dynamic variation (Lin *et al.* 2020), coexpression modules with a power of 15 and merCutHeight of 0.25 were identified. The membership (*K*_ME_) >0.85 was used as a standard of gene connectivity to select the DEGs related to FS. Next, the obtained DEGs that were strongly correlated in specific modules were compared with other DEGs obtained in the physical location corresponding to the marker of QTLs. GO enrichment analysis was performed on these common genes.

### Confirmation of RNA-Seq results by qRT-PCR

The total RNA was extracted from all fiber samples (PimaS-7, 5917, 4 high-FS, and 4 low-FS materials at 0–35 DPA). Primer Premier v.5 was used to design related gene primers. All primers were synthesized by BGI (Beijing, China). Reverse transcription was performed using a Revert Aid First Strand cDNA Synthesis kit (Thermo Scientific, Waltham, MA, USA). qRT-PCR was performed using a Trans Start Top Green qPCR Super Mix kit (Transgen Biotech, Beijing, China). The cotton UBQ7 served as the reference gene to normalize the relative expression levels through ABI 7500 Fast Real-Time PCR System (Applied Biosystems, Waltham, MA, USA), with 3 biological repeats and 3 technical repeats. The 2^−^^ΔΔCT^ method was used to analyze the relative expression of DEGs (Livak and Schmittgen 2001).

## Results

### Physical location of 3 QTLs related to FS

In Fan’s study, a high-density genetic map was constructed using an *F*_2:6_ RIL population (143 lines generated from PimaS-7 × 5917) (Supplementary Tables 1–3). Based on the scaffold map of *G. barbadense* (Yuan *et al.* 2015), 3 QTLs (*qFS-LG21-1*, *qFS-LG1-1*, and *qFS-LG4-1*) related to FS were identified (Fan *et al.* 2018). To further discover candidate genes related to FS, the confidence intervals of the 3 QTLs were compared in the reorganized *G. barbadense* physical map in this study. *qFS-LG21-1*, with a limit of detection (LOD) of 3.38, explains 8.86% of the phenotypic variations, and the confidence interval located at D03:3270717–3902216 covers a physical interval of 0.6 Mb. *qFS-LG1-1*, with an LOD of 3.92, explains 10.42% phenotypic variations, and the confidence interval located at D08:38457462–63870127 covers a physical interval of 24.24 Mb. *qFS-LG4-1*, with an LOD of 4.84, explains 10.11% phenotypic variations, and the confidence interval located at D11:30147612–6797464 covers a physical interval of 36.07 Mb (Supplementary Table 4). According to the *G. barbadense* reference genome, 2,601 genes from D03, D08, and D11 were related to FS (Supplementary Table 5).

### RNA-seq and quality assessment

**Fig. 1.**
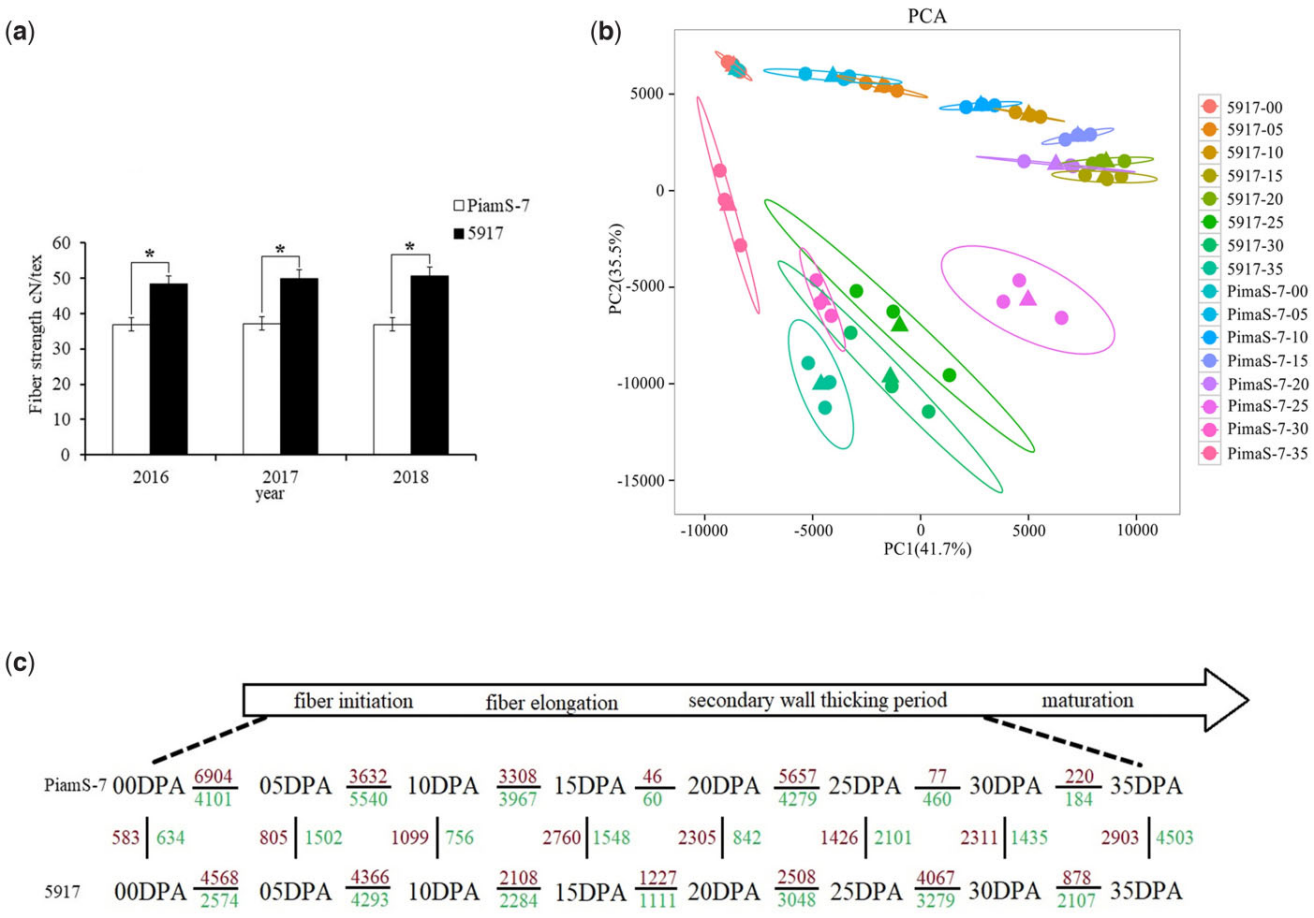
Basic analysis of the PimaS-7 and 5917. a) FS data analysis between PimaS-7 and 5917. The 2 materials had significant differences in FS from 2016 to 2018 (*mean *P* < 0.05). b) PCA between PimaS-7 and 5917 in different fiber development times. c) Comparison of DEGs between PimaS-7 and 5917 in different fiber development times. Horizontal comparisons represent different developmental stages of the same gene. Vertical comparison represents the same developmental stage of different genes. Red represents upregulated genes; blue represents downregulated genes.

To accurately discover genes related to FS, PimaS-7 and 5917 (Fig. 1a) were used as experimental materials for RNA-seq in 8 different fiber development times with 3 biological replications. Forty-eight cDNA libraries were constructed, and 317.57 Gb of clean data was obtained after screening the raw data. The average clean data of each sample reached 5.78 Gb. The percentage of Q30 base was above 92.78%, and the range of GC content was between 44.17% and 46.54% (Supplementary Table 6). The mapping rate between the clean reads of samples and the designated reference genome of *G. barbadense* was 85.56–94.79%. In total, 69,338 genes were considered expressed genes with FPKM > 0.5. Pearson’s correlation coefficient was used to evaluate the correlation among the 3 biological replicates of each sample in the same fiber development times (Supplementary Fig. 1). Then, principal component analysis was used to clarify the relationship between the 2 materials in different fiber development times (Fig. 1b). In the early stages of fiber development, the 2 materials aggregate together. However, with fiber development, the degree of polymerization of the 2 gradually decreased in different fiber development times. The results indicated that the influence of different development times of fiber between the 2 materials was greater than the background difference between them.

### Analysis of DEGs

In the present study, DEGs were obtained by comparing the same materials in different fiber development times and the same fiber development times of different materials. A total of 38,798 genes were selected as DEGs form all express genes with an FDR value ≤0.05 and |Log2(FC)| ≥ 1. In PimaS-7, the DEGs between 00 and 05 DPA were highest in the early fiber development stage; 1,217 DEGs were found (6,904 upregulated, 4,101 downregulated). As fiber development progresses, the DEGs gradually decreases until between 20 and 25 DPA; then, DEGs peaked again, and 9,936 DEGs were found (5,657 upregulated, 4,279 downregulated). In 5917, the number of DEGs between 05 and 10 DPA was the highest in the early fiber development stage, and 8,659 DEGs were found (4,366 upregulated, 4,293 downregulated). After that, 7,346 DEGs were found in 25 and 30 DPA (4,067 upregulated, 3,279 downregulated). PimaS-7 had 1,217 DEGs on 00 DPA, 2,307 DEGs on 05 DPA, 1,855 DEGs on 10 DPA, 4,308 DEGs on 15 DPA, 3,147 DEGs on 20 DPA, 3,527 DEGs on 25 DPA, 3,746 DEGs on 30 DPA, and 7,407 DEGs on 35 DPA (Fig. 1c). These numbers showed that the number of DEGs changed with different fiber development times. At the beginning of the fiber elongation period until the secondary wall-thickening stage, the number of DEGs gradually increased, and it peaked at the end of the secondary wall-thickening stage. In a comparison of gene expression levels between PimaS-7 and 5917 in different fiber development times, many genes were identified as DEGs, and the total number of DEGs of PimaS-7 was similar with that of 5917. The results showed that the difference in FS was due to the gene expression levels in the 2 materials. In previous studies, according to the expression level of genes in different periods and changes in expression patterns, various genes were identified to be related to genetic diseases, yield, and resistance (Zhang *et al.* 2017; Cheng *et al.* 2020; Zhao, Bai, *et al.* 2020).

### Construction of coexpression modules using WGCNA

**Fig. 2.**
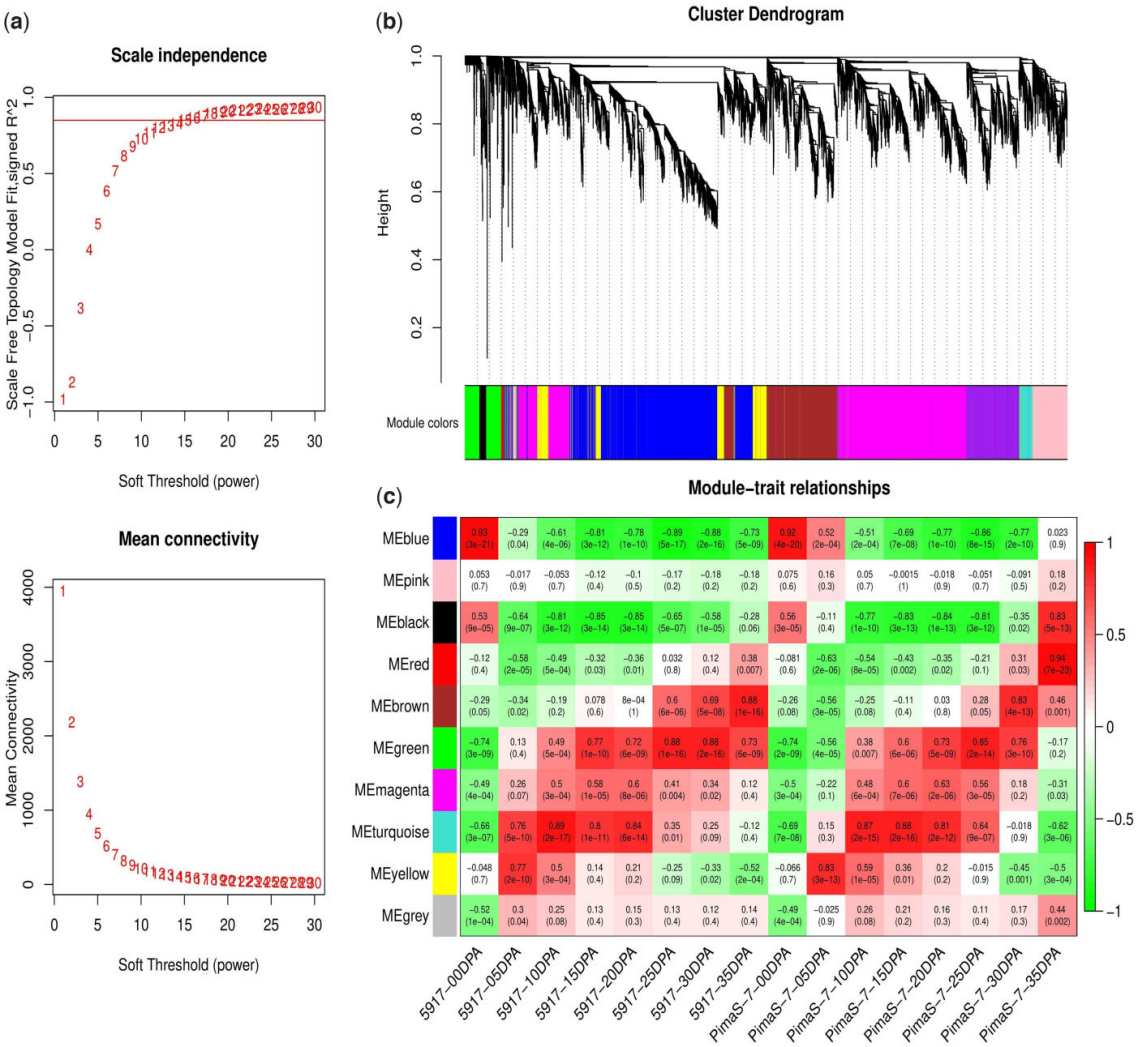
Weighted coexpression network analysis of DEGs in PimaS-7 and 5917. a) Determination of soft threshold of weighted gene coexpression network. b) Hierarchical cluster diagram of coexpression modules identified by WGCNA. Each analysis represents a module, and each leaf represents a gene. c) Module related to the sample. The horizontal axis represents the different development times of the 2 materials, and the vertical axis represents the feature vectors of each module. The redder the color of the module, the higher the correlation with the corresponding fiber development stage.

To further clarify the relationship between the gene expression of the 2 materials in different fiber development times and FS, coexpression networks of DEGs were constructed using WGCNA with a power of 15 (Fig. 2a). Ten modules were identified to be related to different fiber development times; some were correlated with 1 time, while the others were correlated with more than 1 (Fig. 2b). The secondary wall-thickening stage was significantly related to the formation of FS. Then, analyzing the correlation between the modules and different fiber development times, it was found that 4 specific modules were significantly related to 25, 30, and 35 DPA during the secondary wall-thickening stage. The brown module contains 1,278 genes, which were significantly correlated with 5917-35 DPA (*r*^2^ = 0.88, *P *=* *1e–16) and PimaS-7-30 DPA (*r*^2^ = 0.83, *P *=* *4e–13) (Fig. 2c). GO terms were enriched in plant-type secondary cell wall biogenesis, glucuronoxylan glucuronosyltransferase activity, and plasma membrane (Supplementary Table 7). The green module contains 522 genes, which were highly associated with 5917-25 DPA (*r*^2^ = 0.88, *P *=* *1e–16), 5917-30 DPA (*r*^2^ = 0.88, *P *=* *2e–16) and PimaS-7-25 DPA (*r*^2^ = 0.85, *P *=* *2e–14). GO terms were enriched in steryl-sulfatase activity, cytoplasm and proteasome-mediated ubiquitin-dependent protein catabolic process. The MEred module contains 41 genes, which were significantly correlated with PimaS-7-35 DPA (*r*^2^ = 0.94, *P *=* *7e–23). GO terms were enriched in cell periphery, response to unfolded protein and unfolded protein binding. The MEblack module contains 109 genes, which were significantly related with PimaS-7-35 DPA (*r*^2^ = 0.83, *P *=* *5e–13). GO terms were enriched in structural constituents of ribosomes, Golgi-associated vesicle membrane and pectic galactan metabolic process. KEGG pathway analysis was used to further explore the 4 specific modules. MEbrown was enriched in amino sugar, nucleotide sugar metabolism, and phagosome (Supplementary Table 8). MEgreen was enriched in pentose phosphate pathway and carbon metabolism. MEred was enriched in protein processing in endoplasmic reticulum, while MEblack was enriched in ribosome. This shows that DEGs from different modules may be involved in different metabolic pathways to regulate the development of FS.

### Colocalization by QTLs and RNA-seq for the screening of related genes

**Fig. 3.**
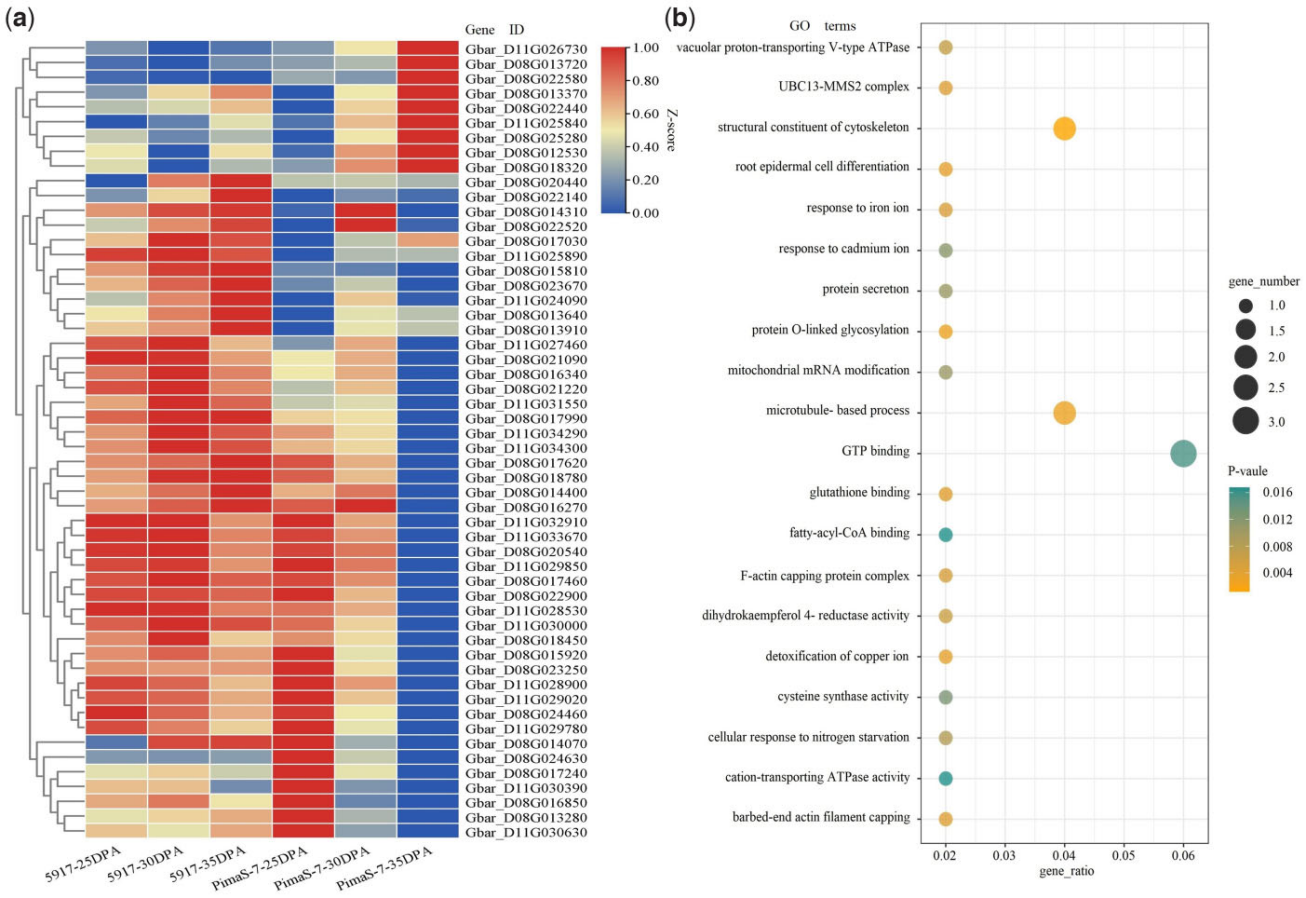
Analysis of the 55 DEGs related to FS. a) Heat map of RNA-seq data of 55 DEGs in 25 DPA, 30 and 35 DPA. Red means high expression. b) GO enrichment analysis of 55 DEGs. The importance of the GO term increases as the *P*-value decreases, gene_number represents the number of genes enriched in each term.

To further narrow down the screening range of candidate genes related to FS, the expression data of 2,601 genes were extracted from RNA-seq data, of which 1,268 genes were identified as DEGs. This DEGs were used for further analysis (Supplementary Table 9). Then, *K*_ME_ > 0.85 was used as a standard of gene connectivity to select DEGs in the 4 specific modules; 1,537 DEGs were considered to participate in the regulation of FS in the 4 modules (MEbrown, MEgreen, MEred, and MEblack; Supplementary Table 10). Finally, a total of 55 DEGs were obtained by comparing them in 2 ways (Supplementary Table 11), and genes showed different expression levels in the secondary wall-thickening stage (Fig. 3a). GO enrichment analysis was performed on these DEGs (Fig. 3b); structural constituents of cytoskeletons, glutathione binding, and GTP binding were enriched in molecular function. F-actin capping protein complex, chloroplast and microtubule were enriched in cellular components. Microtubule-based process and ubiquitin-dependent protein catabolic process were enriched in biological process. Based on GO enrichment analysis, many DEGs mainly concentrated in GO term related to cytoskeletal structures and microtubules. Therefore, the 5 DEGs included in the above pathway as representative genes of different terms were selected for further research. *Gbar_D08G020540* and *Gbar_D11G032910* were selected from the MEbrow module; these were significantly related to fiber development in 30 and 35 DPA. *Gbar_D08G013370* was selected from the MEred module, and it was related to fiber development in 35 DPA. *Gbar_D11G033670* and *Gbar_D11G029020* were selected from the MEgreen module, which were significantly related to fiber development in 25 and 30 DPA.

### Confirmation of RNA-seq results by qRT-PCR

**Fig. 4.**
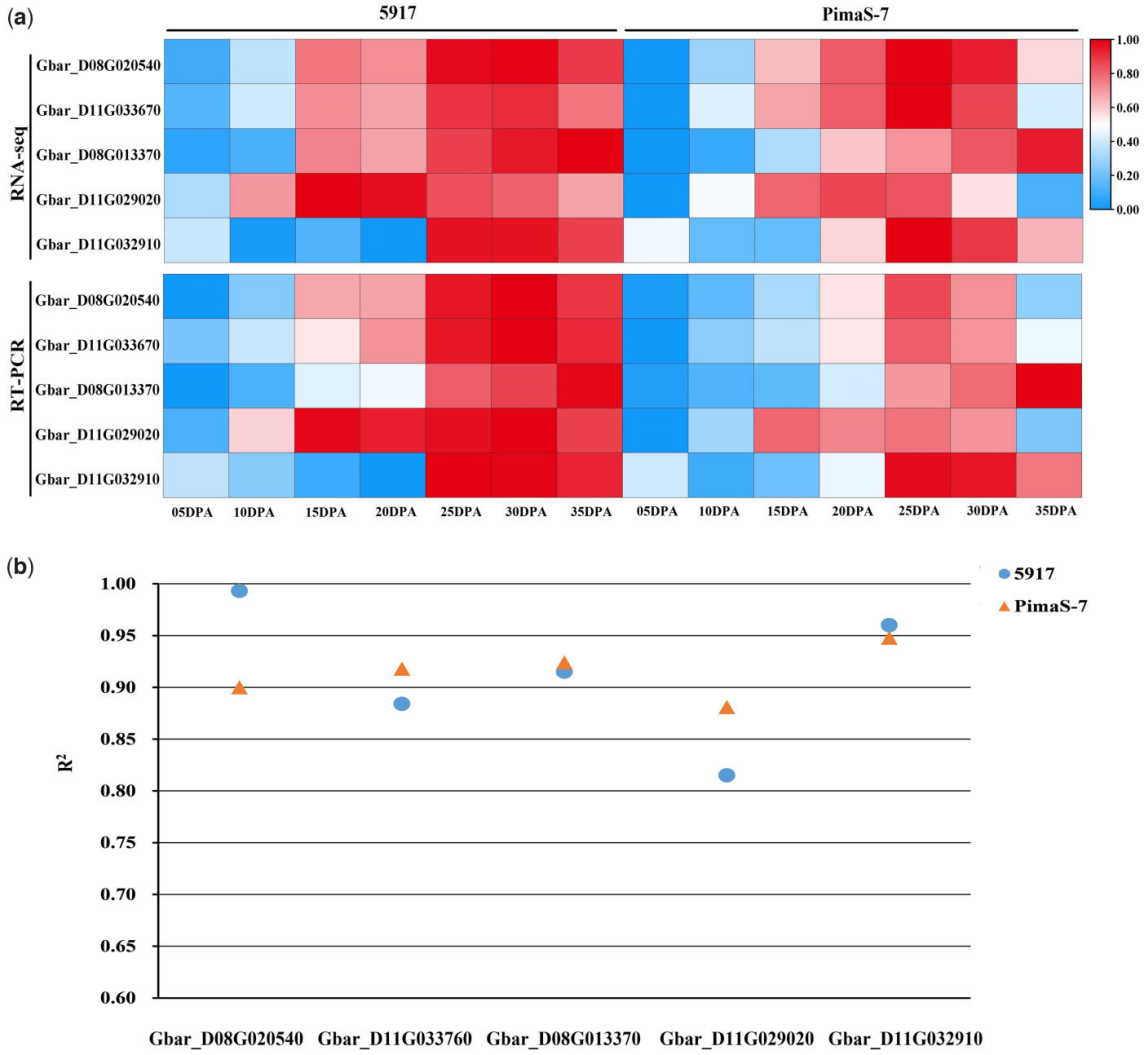
Confirm of expression level of 5 DEGs by qRT-PCR. a) The heat map of qRT-PCR results and RNA-seq data of 5 DEGs in PimaS-7 and 5917. b) Linear regression analysis between qRT-PCR results and RNA-seq data of 5 DEGs. The *R*^2^ value represents the correlation between the RNA-seq data and qRT-PCR results of the 2 materials. Each times represents the fold-change expression level compared with 00 DPA, that were converted by log2 standardization.

qRT-PCR was performed to confirm the RNA-seq data of 5 DEGs (Fig. 4a; Supplementary Table 12). The qRT-PCR results showed that the expression trends of *Gbar_D11G032910*, *Gbar_D08G020540*, *Gbar_D11G033670*, *Gbar_D08G013370*, and *Gbar_D11G029020* in PimaS-7 and 5917 were similar with the transcriptome. The result of linear regression analysis was used to prove that the qRT-PCR results and RNA-seq were significantly correlated (Fig. 4b).

To further confirm the expression patterns of the 5 DEGs, 4 high-FS materials, and 4 low-FS materials were selected from the *G. barbadense* accessions (Table 1), and qRT-PCR was performed (Supplementary Fig. 2). During 0–15 DPA, the expression patterns of 8 materials were similar to those of the 2 parents. In 20 DPA, the upregulation of 4 gene expression levels in high-FS materials gradually became greater than those in low-FS materials. At 25–30 DPA, the expression of the 5 DEGs in all materials showed a rapid upward trend as a whole, and the rate of increase in gene expression level was increased in high-FS materials relative to that in the other group. In 35 DPA, the gene expression level of high-FS materials continued to grow, whereas that of the other group decreased. This indicates that during the secondary wall-thickening stage, the expression trends of the 5 DEGs were similar to those of the 2 parents, and the main difference lies on the difference in gene expression levels.

**Table 1.** The list of fiber quality traits of *G. barbadense* accessions.

Name	Fiber length	Fiber strength	Micronaire	Fiber elongation	Fiber uniformity	Fiber maturity	Spinning consistence index
Xinhai30	33.97 ± 0.62	52.25 ± 2.74	4.22 ± 0.26	5.50 ± 0.43	87.71 ± 0.67	0.87 ± 0.00	219.32 ± 16.76
Xinhai31	33.63 ± 0.52	52.16 ± 2.57	4.65 ± 0.04	5.49 ± 1.03	88.24 ± 0.68	0.88 ± 0.00	222.61 ± 8.49
SI	34.13 ± 1.42	52.15 ± 5.27	4.46 ± 0.21	4.90 ± 0.67	87.91 ± 1.07	0.88 ± 0.00	221.77 ± 20.00
C6015	34.82 ± 1.27	50.56 ± 3.83	4.59 ± 0.06	4.96 ± 0.86	87.55 ± 1.70	0.88 ± 00.00	213.56 ± 14.84
65-3049-6	32.06 ± 0.64	31.09 ± 5.34	4.22 ± 0.48	7.79 ± 0.78	85.54 ± 0.28	0.84 ± 0.00	154.05 ± 20.77
65-3080-2	37.93 ± 0.68	33.83 ± 3.21	3.08 ± 0.22	5.66 ± 0.73	84.65 ± 0.87	0.83 ± 0.00	178.05 ± 5.68
Ashmont	25.05 ± 1.95	29.66 ± 2.08	5.44 ± 0.58	8.25 ± 1.32	81.38 ± 0.60	0.87 ± 0.01	100.32 ± 11.13
75-86	33.98 ± 0.65	29.94 ± 1.30	4.23 ± 0.41	5.75 ± 0.95	84.64 ± 0.86	0.86 ± 0.00	146.08 ± 2.95

### Correlation between the expression levels of the 5 DEGs and FS in *G. barbadense* accessions

To study the relationship between the expression levels of the 5 DEGs and FS, correlation analysis was performed on 8 materials at different fiber development times and FS (Table 2). At 5–20 DPA, there was no correlation between the expression levels of 4 DEGs (*Gbar_D11G033670*, *Gbar_D08G020540*, *Gbar_D11G029020*, and *Gbar_D08G013370*) in *G. barbadense* accessions and FS data, except for *Gbar_D11G032910*. This was consistent with the RNA-seq results, where the DEG expression level was low in the early stages of fiber development. Until the secondary wall-thickening stage, 5 DEGs were positively correlated in *G. barbadense* accessions with FS data. This result is consistent with the related gene expression levels, which showed a rapid increase at 25–30 DPA in the RNA-seq data. Therefore, the high expression of the 5 DEGs may be involved in regulating FS during the secondary wall-thickening stage.

**Table 2.** Correlation analysis of gene expression level and FS of *G. barbadense* accessions with 5 DEGs.

	Gbar_D08G013370	Gbar_D11G029020	Gbar_D11G032910	Gbar_D11G033670	Gbar_D08G020540
05 DPA	0.111	0.196	0.524	0.317	0.445
10 DPA	0.240	0.622	0.297	0.090	0.648
15 DPA	0.341	0.668	0.876**	−0.240	0.135
20 DPA	0.406	0.701	0.897**	0.047	0.229
25 DPA	0.851**	0.840**	0.907**	0.366	0.748*
30 DPA	0.920**	0.938**	0.807*	0.819*	0.868**
35 DPA	0.904**	0.975**	0.912**	0.808*	0.945**

*Correlation is significcnt at 0.05 level (2-tailed). **Correlation is significcnt at 0.01 level (2-tailed)

## Discussion

### Combined analysis of QTL mapping and RNA-seq enables the study of FS in secondary wall-thickening stage

With continuous improvements in the sequencing of the cotton genome, many important genes related to cotton quality are being discovered. Transcriptome technology is widely used to study fiber quality traits in cotton (Lee *et al.* 2007; Yoo and Wendel 2014; Naoumkina *et al.* 2015; Li, Wang, *et al.* 2017) and it further combines QTL, GWAS, TWAS, and eQTL networks to provide more strategies for the improvement of fiber length and FS (Li, Cheng, *et al.* 2018; Li *et al.* 2020). Based on the RNA-seq data of the near-isogenic lines NMGA-062 and NMGA-105 combined with QTL mapping, the candidate genes were finally determined (Li, Wu, *et al.* 2017). Kashif Shahzad used *G. hirsutum* inbred lines and hybrids as materials; RNA-seq, QTL, and gene coexpression network analyses were performed, and *Gh_A03G1024*, *Gh_D08G1440*, and *Gh_A08G2210* were determined as candidate genes (Shahzad *et al.* 2020). Therefore, in the present study, the combined analysis of QTL and RNA-seq was selected to explore the genes associated with FS. In addition, numerous existing studies on fiber quality traits were mostly based on the exploration of fiber cell protrusion and fiber elongation and usually with the *G. barbadense* fragment inserted in a single background of *G. hirsutum* as the research background (Islam *et al.* 2016). It cannot be ignored that due to the different genetic backgrounds of the 2 genomes, the growth period of *G. barbadense* was longer than that of *G. hirsutum*, and the 4 stages of fiber development were also different in *G. hirsutum* (Gilbert *et al.* 2014). Therefore, most genes identified as candidate genes in different research materials may be due to the differences between the species (Chen *et al.* 2012). The completion of gene sequencing of *G. barbadense* provides an accurate reference genome for the study of FS traits under the background of a single *G. barbadense* (Wang *et al.* 2019). In the present study, 2,601 genes of D03, D08, and D11 were obtained from *G. barbadense* in Fan’s studies. These were believed to be related to FS. Next, transcriptome technology was used to sequence the 48 cDNA libraries with 2 types of materials from *G. barbadense*. After comparing the genes obtained by the 2 methods, a link was found between the results. These combined research methods were conducive for a more comprehensive study of the developmental mechanisms related to FS.

### Analysis of candidate genes related to FS

Functional analysis of related DEGs revealed that many genes were mainly involved in term such as the composition of structural constituents of the cytoskeleton and microtubule-based processes. Therefore, 5 DEGs were selected as representative genes in the main terms and further verified by qRT-PCR. The results of qRT-PCR were consistent with the RNA-seq data, indicating that DEGs located in the main terms have regulatory effects on FS. Previous studies showed that cellulose and pectin are important components of plant cell walls and play an important role in fiber cells (Gilbert *et al.* 2013; Sun *et al.* 2020). Cellulose synthase 6 (*CesA6*) mutants show a strong cellulose deficiency, and hence a short hypocotyl phenotype is observed in *Arabidopsis* (Hematy *et al.* 2007). Therefore, DEGs involved in the regulation of cell wall composition during the secondary wall-thickening stage were more likely to regulate FS.

The main components of cytoskeletons are microtubules and actin. *Gbar_D11G029020* encodes a capping protein with 303 amino acids, which does not depend on Ga^2+^-bound actin. As the main component of microfilaments, actins, like tubulins, regulate the transport of intracellular substances and maintain the cytoskeleton stability (Mathur *et al.* 2003). In upland cotton, *GhXLIM6*, *GhWLIM2*, *GhWLIM5*, and *GhPLIM1* were highly expressed during the secondary cell wall-thickening stage (Zhao *et al.* 2010; Li *et al.*^2013^, ^2015^). When the expression of *GhXLIM6* was inhibited, its association with F-actin in cotton fibers was destroyed, and the actin cytoskeletons were distorted, which seriously affected the formation of fiber length and FS (Li, Wang, *et al.* 2018). Other studies also proved that regulating actin can cause fiber elongation defects and affect microtubules and the direction of cellulose deposition (Li *et al.* 2005; Wang *et al.* 2010; Cao *et al.* 2021). Therefore, the persistent high expression of *Gbar_D11G029020* during the secondary cell wall-thickening stage may help maintain cell wall stability to enhance FS.

*Gbar_D11G033670* and *Gbar_D08G020540* encode proteins with 447 and 444 amino acids, respectively, belonging to the *β*-tubulin group in microtubules. *Gbar_D11G032910* encodes a protein with 360 amino acids that contains a TPX2 domain belonging to microtubule-associated proteins. Microtubules are hollow tubular structures that run longitudinally along the cell wall. They play an important role in biological processes as structural components of the cytoskeleton for vesicle transport and cell wall deposition (Seagull 1998; Sedbrook and Kaloriti 2008). In *Arabidopsis*, *ATWVD2* can interact with microtubules, promote microtubule bundling, and play an important role in polar cell elongation (Perrin *et al.* 2007). The overexpression of *ATWDL3* and RNAi-downregulated lines showed shorter and longer hypocotyl cells than the wild-type, respectively (Liu *et al.* 2013). Previous research on microtubules genes in cotton mainly focused on fiber quality in the early stage of fiber development in *G. hirsutum* (Li, Wu, *et al.* 2017; Qin *et al.* 2019; Zou *et al.* 2019). Research on microtubules is gradually increasing. Microtubule genes are deemed to have specific expression levels in different tissues and organs (Tuttle *et al.* 2015), and multiple microtubule genes are highly expressed during fiber development and are regulated by hormones (Islam *et al.* 2016). *GhWDLA7* possibly regulates FS through its interaction with *GhTUA2* (Lei *et al.* 2019).

*Gbar_D08G013370* encodes the protein SPIRAL1-like 2. Studies showed that the interaction of SPIRAL1-like 2 and SPIRAL1-like 1 could regulate cortical microtubules required to grow anisotropic cells (Nakajima *et al.* 2006). Cellulose synthase interactive protein 1 (encodes by *CSI1*) connects the cellulose synthase in vesicles to cortical microtubules (Li *et al.* 2012). At the same time, neatly arranged cortical microtubules under the plasma membrane guide and regulate the synthesis direction and deposition rate of cellulose fibrils on the plasma membrane (Gutierrez *et al.* 2009; Zhong and Ye 2015). When the genes involved in cell wall rearrangement were affected, the transcription of cellulose synthase was inhibited (Gilbert *et al.* 2013). From this, we can speculate that the orderly arrangement of cortical microtubules not only affects the cytoskeletal structures, but also affects the deposition of cellulose. Therefore, the continuous high expression of representative DEGs in main terms such as cytoskeletal structures and microtubules in high-FS materials provide a direction for the study of FS. Although numerous existing studies could prove that the current cellulose synthase and microtubule candidate genes were able to affect the formation of FS by regulating the pathways of fiber formation, it is necessary to further explore the specific positions of different genes in the pathways.

## Conclusion

In summary, based on previous studies, 3 QTLs related to FS were relocated. The RNA-seq method was used to study the differences in gene expression at different fiber development stages between the 2 parents (PimaS-7 and 5917). Forty-eight cDNA libraries were constructed using RNA-seq, containing 2 materials and 3 biological replicates. Four specific modules closely related to the secondary wall-thickening stage were obtained using WGCNA. In genetic colocalization using QTL and RNA-seq, 55 candidate genes were identified. GO enrichment analysis showed that many DEGs mainly concentrated in GO term related to cytoskeletal structures and microtubules. The qRT-PCR results showed that the expression levels of the 5 representative genes in the main terms were significantly higher than those in low-FS materials during the secondary wall-thickening stage of fiber development. Results showed that *Gbar_D11G032910*, *Gbar_D08G020540*, *Gbar_D08G013370*, *Gbar_D11G033670*, and *Gbar_D11G029020* may regulate FS by playing a role in the composition of structural constituents of the cytoskeleton during fiber development.

## Data availability

Supplementary Fig. 1 contains Pearson’s correlation coefficient analysis of population parents. Supplementary Fig. 2 contains 5 DEGs were confirmed by qRT-PCR of *G. barbadense* accessions. The linkage map, RIL genotype, and RIL phenotype mean for fiber traits in Supplementary Tables 1–3. Supplementary Table 4 contains the marker intervals and the physical confidence intervals of the QTLs related FS. Supplementary Table 5 contains 2,601 genes were included in 3 QTLs physical position. Supplementary Table 6 contains summary of RNA-seq data. The RNA-seq raw data underlying this article are available in NCBI database with the accession number: GSE178945, at https://www.ncbi.nlm.nih.gov/geo/query/acc.cgi?acc=GSE178945. Supplementary Table 7 contains GO-term enrichment of 4 significant WGCNA modules. Supplementary Table 8 contains KEGG pathway enrichment of 4 significant WGCNA modules. Supplementary Table 9 contains 1,268 different expression genes were included in 3 QTLs physical position. Supplementary Table 10 contains the list of gene in 4 specific modules. Supplementary Table 11 contains description of 55 colocalized differentially expressed genes. Supplementary Table 12 contains primer sequences used in qRT-PCR.

Supplemental material is available at *G3* online.

## Supplementary Material

jkac167_Figure_S1

jkac167_Figure_S2

jkac167_Table_S1

jkac167_Table_S2

jkac167_Table_S3

jkac167_Table_S4

jkac167_Table_S5

jkac167_Table_S6

jkac167_Table_S7

jkac167_Table_S8

jkac167_Table_S9

jkac167_Table_S10

jkac167_Table_S11

jkac167_Table_S12
