# Supplementary material for: Analysis of transcriptome data and quantitative trait loci enables the identification of candidate genes responsible for fiber strength in Gossypium barbadense
Source: G3 (Bethesda). 2022 Jul 26;12(9):jkac167. doi: 10.1093/g3journal/jkac167 (PMC9434320; doi:10.1093/g3journal/jkac167)
Supplement: jkac167_Figure_S1 [file jkac167_figure_s1.pdf]

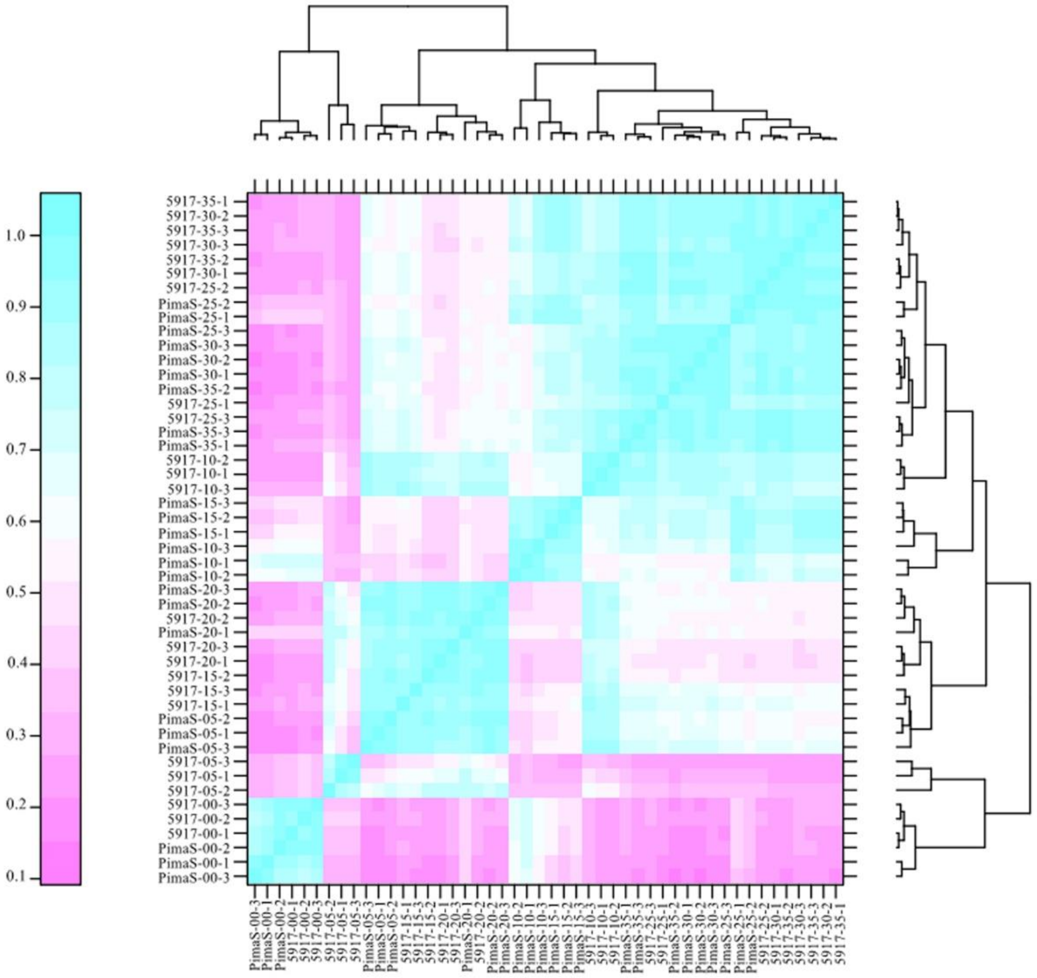

Figure S1. Pearson correlation coefficient analysis of RNA-seq data from fiber tissue of PimaS-7 and 5917 in different fiber development times.
