# Supplementary material for: Analysis of transcriptome data and quantitative trait loci enables the identification of candidate genes responsible for fiber strength in Gossypium barbadense
Source: G3 (Bethesda). 2022 Jul 26;12(9):jkac167. doi: 10.1093/g3journal/jkac167 (PMC9434320; doi:10.1093/g3journal/jkac167)
Supplement: jkac167_Figure_S2 [file jkac167_figure_s2.pdf]

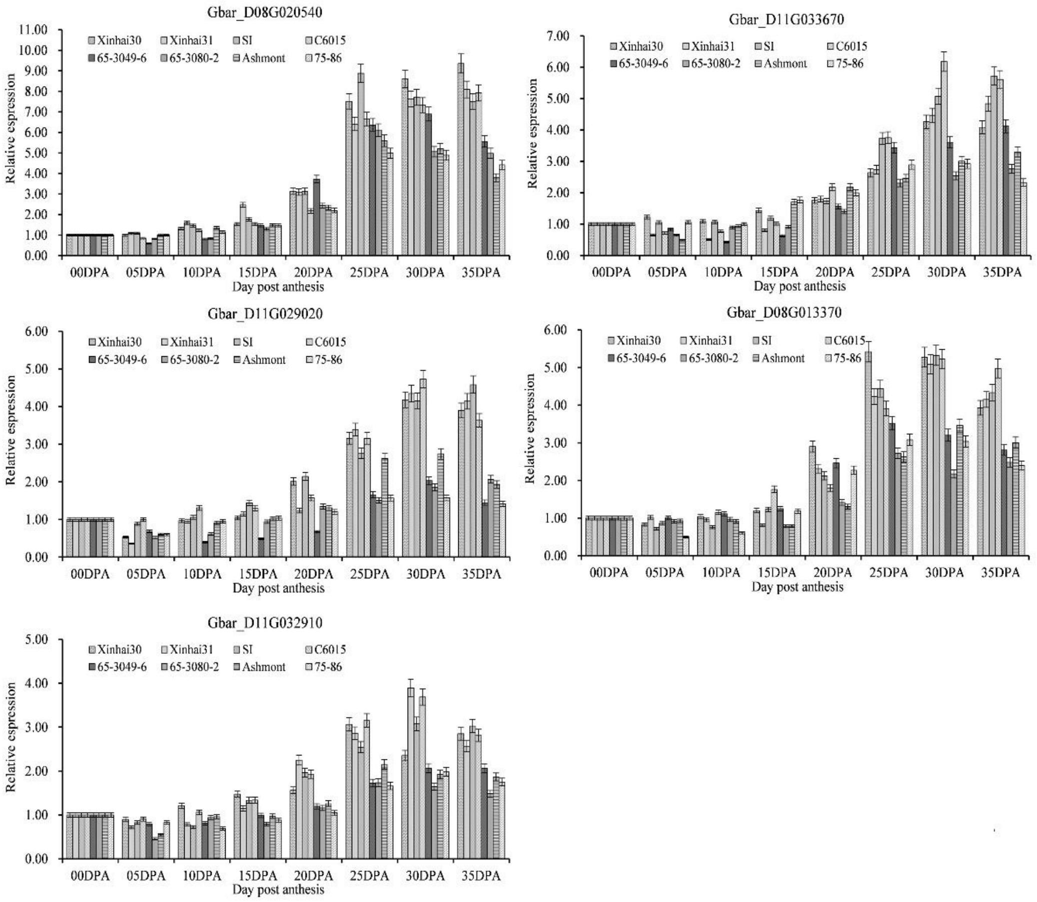

Figure S2. The expression patterns of five genes in eight *G. barbadense* accessions were further validated using qRT-PCR.
